# Oral Health Status Among Children and Adolescents from Vulnerable Populations: A Cross-Sectional Study in Seville, Spain

**DOI:** 10.3390/dj13110522

**Published:** 2025-11-07

**Authors:** Rodolfo Esteban Reyes-Lara, Adrián Curto, David Ribas-Perez, Ignacio Barbero-Navarro, Diego Rodriguez-Menacho, Javier Flores-Fraile, Antonio Castaño-Séiquer

**Affiliations:** 1 Department of Surgery, Faculty of Medicine, University of Salamanca, 37007 Salamanca, Spain; rodolfoestebanreyeslara@gmail.com (R.E.R.-L.); adrian_odonto@usal.es (A.C.); ibarbero2@us.es (I.B.-N.); drmenacho@us.es (D.R.-M.); acastano@us.es (A.C.-S.); 2 Department of Stomatology, Faculty of Dentistry, University of Seville, 41005 Seville, Spain

**Keywords:** oral health, dental caries, children, adolescents, social inequalities

## Abstract

**Background/Objectives**: The aim of this study was to evaluate the oral health status of socially vulnerable children and adolescents in Seville and to examine its associations with behavioral and sociodemographic determinants. We hypothesized that greater social vulnerability and suboptimal behaviors would be associated with higher caries experience. **Methods**: A cross-sectional analytical study was conducted on 250 participants aged 2 to 17 years attending the Luis Séiquer Social Dentistry Foundation between January and March 2025. Clinical examinations followed the WHO Oral Health Surveys: Basic Methods (5th edition, 2013) and were performed by a calibrated dentist. Variables included dental caries indices (dft, DMFT), pulpal treatment needs, and oral hygiene practices. Statistical analyses included Kruskal–Wallis, Mann–Whitney U, Fisher’s exact tests, and multivariate regression models (significance level *p* < 0.05). **Results**: Caries prevalence was high across all age groups, particularly in primary dentition (mean dft = 3.05 ± 3.80; DMFT = 2.99 ± 3.66; *p* < 0.001). Pulp therapy needs were significantly higher among preschoolers (mean = 2.22 ± 2.31). Factors such as low parental education, migrant background, insufficient toothbrushing frequency, and frequent sugar intake were strongly associated with poorer oral health outcomes. Although fluoridated toothpaste use was widespread (>94%), dental floss use remained limited (34.8%). Multivariate analyses confirmed a strong association between social inequalities and oral disease burden. **Conclusions**: Children and adolescents from vulnerable groups in Seville experience a high prevalence of dental caries and substantial unmet treatment needs. Findings highlight marked oral health disparities linked to socioeconomic status, emphasizing the urgent need for early preventive programs, culturally adapted oral health education, and equitable access to dental care.

## 1. Introduction

Oral health represents a fundamental component of overall health and well-being, playing a pivotal role in human development from early childhood through adolescence. Its relevance extends beyond the mere absence of oral disease, exerting significant reciprocal effects on nutrition, communication, cognitive and academic performance, self-esteem, and, ultimately, quality of life at both individual and societal levels [1,2].

Globally, the World Health Organization (WHO), through its Global Oral Health Strategy and Action Plan 2023–2030, underscores the substantial burden of oral diseases, which affect billions of people worldwide and impose considerable social and economic consequences. The strategy advocates for a paradigm shift toward prevention, integration of oral health within primary care, and improved equitable access to dental services [3]. At the national level, reports such as the Spanish Oral Health Barometer [4] and findings from the Spanish Dental Council [5] highlight persistent challenges in addressing oral health inequalities.

Despite advances in preventive strategies, dental caries remains the most prevalent chronic disease in childhood and adolescence [6,7,8]. These figures reveal a markedly unequal distribution, disproportionately affecting socially and economically disadvantaged populations [9]. This persistent inequality is a global phenomenon [10]. From an epidemiological perspective, determinants such as low socioeconomic status, restricted access to dental services, limited health education, and inadequate behavioral habits are well-documented modulators of oral health outcomes in these vulnerable groups [11,12]. International studies have confirmed that social inequality—as measured by family income and parental education—is a strong predictor of childhood caries across developed countries [13].

Factors including parental education, family environment, migrant status, and economic and geographic barriers to care exert a direct influence on the onset and severity of oral pathologies [14]. In vulnerable populations, these inequities are exacerbated by environmental and social constraints that hinder preventive practices and limit access to timely treatment [5,11].

The consequences of poor oral health during childhood and adolescence extend far beyond the oral cavity. Untreated dental diseases are associated with chronic pain, recurrent infections, masticatory and phonetic impairment, academic underachievement, school absenteeism, and significant psychosocial effects, including reduced self-esteem and social interaction [2,3,4,9]. Given this burden, there is an urgent need for ongoing epidemiological surveillance and the implementation of comprehensive public health strategies aimed at ensuring equitable access to preventive and restorative services [6].

The geographical context of Andalusia, and specifically the province of Seville, presents a scenario of vulnerability that amplifies health inequalities. According to the EAPN Andalucía Report on Poverty 2023, this region has the highest AROPE rate (risk of poverty and/or social exclusion) in Spain, reaching 37.5% of the population (3.2 million people). Alarmingly, the AROPE rate among children and adolescents was 47.1% in 2023, the highest for the fourth consecutive year. This high and increasing rate of poverty, combined with unemployment indicators above the national average in several areas of Seville and the concentration of immigrant populations in specific neighborhoods, creates an environment of exclusion that directly affects access to basic resources and services, including healthcare and oral health promotion [15,16,17,18]. 

Recent Spanish studies have confirmed the role of social and behavioral inequalities in children’s oral health. However, to date, no research has examined these associations in Seville, a province with among the highest poverty and exclusion rates nationally. By addressing this gap, the present study adds original evidence to the existing literature [19,20,21,22].

In this context, the present study aimed to characterize the oral health status of children and adolescents from vulnerable groups in Seville, Spain. We assessed clinical indicators (e.g., dental caries prevalence, periodontal status, and pulp therapy needs), behavioral factors (oral hygiene habits and dietary patterns), and sociodemographic determinants, applying validated epidemiological tools and indices. This integrative approach provides evidence to guide targeted preventive strategies and health policies addressing inequalities in pediatric oral health.

## 2. Materials and Methods

### 2.1. Study Design and Population

A cross-sectional, observational, and analytical study was conducted, in collaboration with the University of Salamanca, to evaluate oral health status and associated factors in a sample of 250 children and adolescents aged 2 to 17 years from socially vulnerable populations in Seville, Spain. Data were collected at the Luis Séiquer Social Dentistry Foundation between January and March 2025.

The minimum sample size was calculated using the formula for cross-sectional prevalence studies: *n* = Z^2^ × *p* × (1−*p*)/d^2^. Assuming a caries prevalence of 50% (*p* = 0.50), 95% confidence (Z = 1.96), and 7% precision (d = 0.07), the required sample size was 196 children. A total of 250 participants were examined, exceeding the minimum required. It should be noted that the children involved in the examiner calibration process were not included in the final analytical sample.

### 2.2. Eligibility Criteria

Children were referred to the Fundación Luis Seiquer by community social services or NGOs, which certified situations of vulnerability based on indicators consistent with the AROPE criteria. The condition of ‘socially vulnerable group’ was accredited through formal referral to FOS by either the Community Social Services of the Seville City Council or Collaborating Third Sector Social Action Organizations (e.g., Cáritas Diocesana, Red Cross, Save the Children, or associations working with immigrants and refugees). These bodies certify the families’ situation of vulnerability or risk of exclusion, which is generally defined by the presence of one or more of the following indicators, in line with the AROPE criteria [17]:

- Risk of severe poverty or severe material deprivation: Household income below the extreme poverty threshold.
- Very low work intensity within the household.
- Complex family situation: single-parent families with low income; large families with all members unemployed; immigrant families in an irregular administrative situation or recent arrivals.
- Recipients of social aid programs: families receiving the Minimum Vital Income (IMV) or the Minimum Social Insertion Income of Andalusia (RMISA).
- Children or adolescents eligible for free school meal grants due to socioeconomic reasons.

Inclusion criteria covered children aged 2–17 years from families at risk of poverty, low work intensity, single-parent or migrant households, or receiving social aid; belonging to families with low household income or unemployment; from recent immigrant families or single-parent households; and in attendance at schools or community centers affiliated with the foundation. Exclusion criteria included refusal of parental consent and the presence of systemic conditions requiring special dental care. The study followed a convenience sampling approach, targeting socially vulnerable groups as defined by the foundation, and was selected by convenience sampling among those referred during the study period.

### 2.3. Clinical Examination

Oral examinations were performed following the World Health Organization (WHO) Oral Health Surveys: Basic Methods, 5th Edition [23,24,25]. All assessments were conducted by a single calibrated dentist using a head-mounted LED light, a plane dental mirror, and a WHO periodontal probe. The examiner underwent a calibration process using a sample of 30 children not included in the final analysis. Intra-examiner reliability was assessed by duplicate examinations two weeks apart. Agreement levels were quantified using the Cohen’s Kappa coefficient for categorical variables (caries diagnosis, treatment needs, pulp therapy indication) and the Intraclass Correlation Coefficient (ICC) for continuous indices (dft/DMFT). The calibration yielded satisfactory agreement values (Kappa = 0.85; ICC = 0.91), ensuring diagnostic consistency.

An intra-examiner calibration process was performed prior to data collection to ensure diagnostic consistency.

The following epidemiological indicators were recorded:

- For caries experience, dft (decayed, filled, and extracted teeth in primary dentition) and DMFT (decayed, missing, and filled teeth in permanent dentition) were recorded as dft in primary teeth and DMFT in permanent teeth. In mixed dentition, both indices were recorded separately, following WHO recommendations.
- Pulp therapy needs were determined based on clinical diagnosis. Pulp therapy needs were determined clinically following WHO and AAPD guidance: deep carious lesions with clinical signs of irreversible pulpitis (spontaneous/nocturnal pain, tenderness to percussion), presence of fistula/abscess, or extensive cavitated lesions with pulp exposure. Radiographs were not systematically available; diagnosis was primarily clinical.
- Oral hygiene practices, including brushing frequency, use of fluoridated toothpaste, and dental floss, were recorded. Toothbrushing frequency was recorded as <1/day, 1/day, 2/day, or >2/day; “infrequent brushing” was defined as ≤1/day.
- Dietary habits, specifically the frequency of free-sugar consumption, were recorded. Free-sugar intake was captured in four categories (≤1/week, 2–4/week, 1/day, and ≥2/day). For regression, it was dichotomized into low (≤1/day) vs. high (≥2/day).

### 2.4. Sociodemographic Variables

Data on age, sex, country of origin, and educational level were obtained via structured interviews with parents or guardians. Data on oral hygiene and dietary habits were collected using structured questionnaires completed by parents/guardians, complemented by self-reports in adolescents aged ≥12 years. Brushing frequency was categorized as “once daily,” “twice daily,” or “more than twice daily.” The use of fluoridated toothpaste and dental floss was recorded as dichotomous variables (yes/no). Sugar consumption was classified as low (≤1 sugary intake per day) or high (>1 per day), based on parental reporting. Sociodemographic information included age, sex, parental educational level (primary, secondary, or higher), family structure, and migration background.

### 2.5. Statistical Analysis

All statistical analyses were conducted using IBM SPSS Statistics for Windows, Version 25.0 (IBM Corp., Armonk, NY, USA). Descriptive statistics included means, medians, standard deviations (SD), and ranges for continuous variables, while categorical variables were summarized using absolute frequencies and percentages. Comparative analyses were performed using the Mann–Whitney U and Kruskal–Wallis H tests for non-normally distributed continuous data, with Scheffé’s post hoc test applied when significant differences were detected. Fisher’s exact test was used to evaluate categorical variables, including pulp therapy needs, sex, country of origin, educational level (school grade), toothbrushing frequency, use of fluoridated toothpaste and dental floss, and sugar consumption frequency. To explore associations and control for potential confounding factors, univariate correlations were calculated using Pearson’s and Spearman’s coefficients, and multivariate linear regression models were applied to assess independent predictors of caries experience and treatment needs. Statistical significance was set at *p* < 0.05.

Country of origin was included as a categorical variable in the multivariate analysis. In SPSS, this variable was automatically dummy-coded, with Spain as the reference group and individual non-Spanish countries represented as separate categories. As a result, some countries with small sample sizes (e.g., Palestine, Gambia, and Ukraine) appeared individually in the regression outputs. We acknowledge that this coding may fragment the data and have addressed this as a limitation in the Discussion.

### 2.6. Ethical Considerations

The study adhered to the Declaration of Helsinki principles and received approval from the Ethics Committee of the University of Salamanca, registration number 1078, on 11/23/2023. Written informed consent was obtained from parents or legal guardians. Participation was voluntary, and confidentiality of all personal data was strictly maintained.

## 3. Results

### 3.1. Sociodemographic Characteristics

The study included 250 participants aged 2 to 17 years (mean age = 10.95 ± 3.99 years), who were stratified into three developmental groups according to dentition stages: Group A (GA) comprised children aged 2–6 years with primary dentition, Group B (GB) included participants aged 7–11 years with mixed dentition, and Group C (GC) consisted of adolescents aged 12–17 years with permanent dentition.

Sex distribution was balanced (49.2% female, 50.8% male). Regarding country of origin, 46.0% were Spanish, followed by 32.4% Latin American, 17.6% African, and 4.4% other nationalities.

Regarding educational level, 50.0% were enrolled in primary school, 21.2% in secondary education, and 20.4% in upper secondary school, while 8.4% attended kindergarten (Table 1).

### 3.2. Caries Indices

#### 3.2.1. Primary Dentition (Dft Index)

The mean dft index was 3.05 ± 3.80 (median = 1.00; range = 0–15). Significant differences were observed across age groups (*p* < 0.001, Kruskal–Wallis test), with the highest values recorded in Group A (2–6 years).

#### 3.2.2. Permanent Dentition (DMFT Index)

The mean DMFT index was 2.99 ± 3.66 (median = 2.00; range = 0–22). Group comparisons showed significant differences between dentition stages (*p* < 0.001, Kruskal–Wallis test).

#### 3.2.3. Pulp Therapy Needs

The mean number of teeth requiring pulp therapy was 0.83 ± 1.54 (median = 0; range = 0–8) (Table 2).

**Figure 1:**
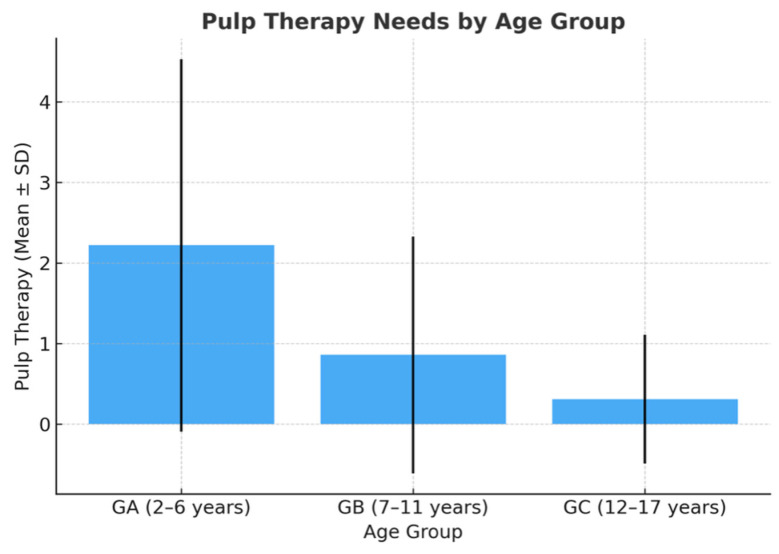
Pulp therapy needs by age group.

Preschoolers (Group A) showed significantly higher treatment needs (mean = 2.22 ± 2.31) compared with Group B (0.86 ± 1.47) and Group C (0.31 ± 0.80) (*p* < 0.001) (Figure 1).

### 3.3. Oral Hygiene Habits

**Figure 2:**
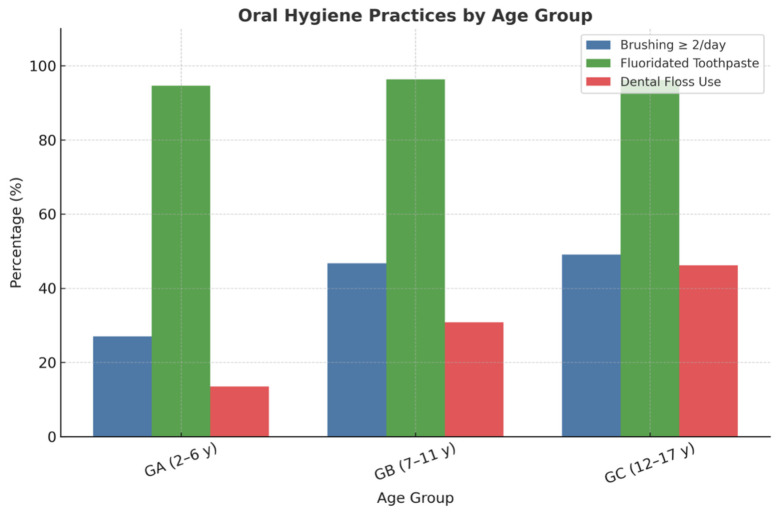
Oral hygiene practices among children and adolescents from vulnerable populations, including toothbrushing frequency, fluoridated toothpaste use, and dental floss utilization.

Regarding oral hygiene practices, most participants reported brushing their teeth twice daily (44.8%), followed by once daily (38.0%) and three times daily (17.2%). The highest prevalence of once-daily brushing was observed among Group A (2–6 years) (64.9%), whereas adolescents in Group C (12–17 years) reported the highest proportion of brushing at least twice daily (49.1%). The use of fluoridated toothpaste was remarkably high across all age groups (>94%), reflecting widespread awareness of its preventive benefits. In contrast, the use of dental floss was notably limited (34.8% overall), with significant differences by age: only 13.5% in Group A compared with 46.2% in Group C. These findings highlight a positive trend toward improved oral hygiene habits among older participants but reveal persistent gaps in the adoption of interdental cleaning practices (Figure 2).

### 3.4. Multivariate Analysis

**Figure 3:**
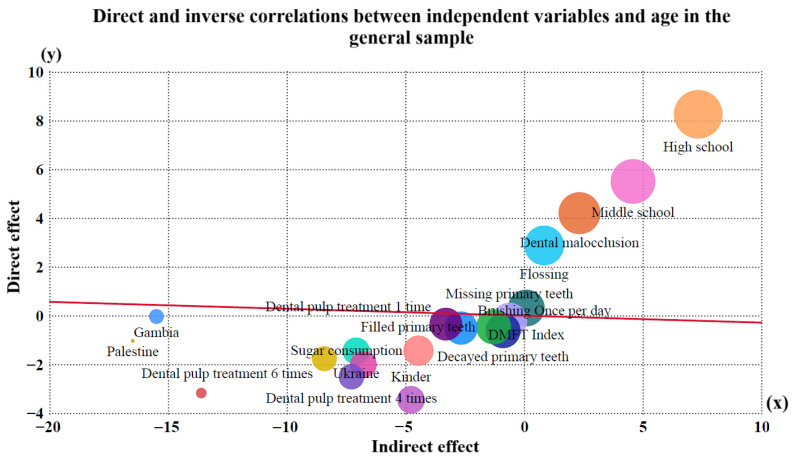
Direct and indirect effects of independent variables on age in the general sample. Bubble size indicates variable influence.

**Figure 4:**
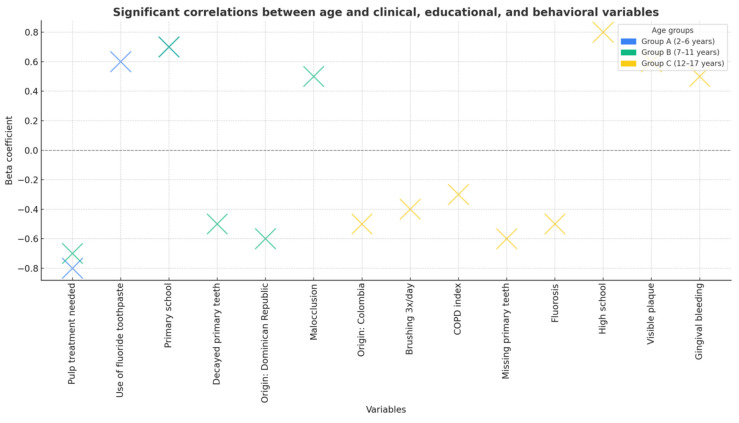
Significant correlations between age and clinical, educational, and behavioral variables across age groups. Beta coefficients indicate the strength and direction of associations.

Multivariate regression analysis revealed significant associations between oral health outcomes and various sociodemographic, behavioral, and clinical factors. Age was negatively correlated with the number of decayed, missing, and filled primary teeth (*p* < 0.001), low educational level, infrequent toothbrushing, high sugar consumption, and pulp therapy needs. Conversely, positive correlations were observed between age and the DMFT index, higher educational attainment, and dental floss use, indicating that older participants tended to present better oral hygiene habits and improved preventive practices despite an increased cumulative caries experience (Figure 3 and Figure 4).

The analysis identified several significant predictors of caries experience in the study population (Table 3). Infrequent toothbrushing (≤1 time/day), frequent sugar consumption, lower parental educational level, and greater pulp therapy needs were associated with higher dft/DMFT scores. Conversely, age was negatively correlated with caries in primary dentition, reflecting the natural exfoliation of deciduous teeth. Children of non-Spanish heritage also showed significantly higher caries indices. The overall model was statistically significant (R = 0.814; adjusted R^2^ = 0.637; *p* < 0.001), explaining 63.7% of the variance in caries outcomes.

## 4. Discussion

This study reveals a high prevalence of dental caries among children and adolescents from socially vulnerable populations in Seville, particularly in primary dentition and in younger age groups. These findings are consistent with prior research conducted in Spain, where caries prevalence in disadvantaged school populations frequently exceeds 70% [24,25], and where significant disparities are observed according to socioeconomic status and migrant background [26,27].

### 4.1. Impact of Migrant Status on Oral Health

In our sample, migrant origin emerged as a significant determinant of poor oral health outcomes. Children from countries such as Gambia, Palestine, and Ukraine exhibited higher dft indices, greater pulp therapy needs, and lower use of preventive practices. These findings align with studies reporting that immigrant children in Spain have higher rates of untreated caries and limited access to dental care services [28,29]. Research conducted in Melilla similarly highlights substantial oral health inequalities and an increased burden of disease among refugee populations [30,31]. This pattern is consistent with other findings in Spain, where parents’ origins and sociocultural inequalities are key determinants of oral health in preschoolers [19].

### 4.2. Educational Level and Health Inequalities

The educational level of both caregivers and children proved to be a key predictor of oral health status. Consistent with previous national and international studies [32,33,34], we observed that children from families with lower educational attainment demonstrated significantly worse oral health outcomes. This supports the widely documented social gradient in oral health [20,35,36], whereby lower educational levels are linked to reduced access to preventive care, limited knowledge of hygiene practices, and higher exposure to risk behaviors.

Our findings also underscore the influence of oral hygiene and dietary habits on caries prevalence and treatment needs. Infrequent toothbrushing and high sugar intake were strongly associated with poor oral health indicators. These results are consistent with evidence showing that behavioral factors are central to the prevention of early childhood caries [37,38,39]. A study in Mallorca, for instance, showed how parents’ education level and the type of residential area influence nutrition habits and, consequently, dental caries in schoolchildren [20].

### 4.3. Behavioral and Dietary Determinants

Our findings underscore the influence of oral hygiene and dietary habits on caries prevalence and treatment needs. Infrequent toothbrushing, high sugar intake, and absence of flossing were strongly associated with poor oral health indicators. In contrast, fluoridated toothpaste use—notably high in this population (>94%)—and flossing were protective factors. Interestingly, only 34.8% of participants reported using dental floss, a gap that could be addressed through targeted educational interventions. These results are consistent with evidence showing that behavioral factors are central to the prevention of early childhood caries [37,38,39]. Moreover, a longitudinal study by Do et al. demonstrated that oral health education fosters resilience and enhances well-being among children from disadvantaged backgrounds [40].

### 4.4. Access to Dental Care Services

Despite partial public coverage, barriers to dental care access persist, especially among migrant families and households with limited financial resources [41]. This structural inequity contributes to the high prevalence of untreated lesions in our sample and mirrors national data, where 33% of Spanish children under six have untreated caries [42]. In addition, poor oral health has been shown to negatively affect self-esteem and quality of life [43], reinforcing the psychosocial impact of oral diseases on child development.

### 4.5. Comparison with European and Global Data

Our results align with findings from European studies demonstrating that caries prevalence among preschool children correlates strongly with socioeconomic inequality [44,45]. At the global level, the WHO emphasizes that tackling oral health inequalities is essential to achieving universal health coverage [46,47,48,49]. Furthermore, recent research in Andalusia highlights that molar-incisor hypomineralization (MIH) and dental caries act as markers of oral health inequality, underscoring the role of social determinants in pediatric oral health outcomes [50,51,52].

### 4.6. Strengths and Limitations

This study provides valuable insight into the oral health of a vulnerable child and adolescent population in Seville; however, its limitations must be acknowledged. The most significant is the use of convenience sampling, a non-probability selection strategy. While this approach was essential to access and characterize a hard-to-reach population facing high social exclusion, it implies that the results are not directly generalizable to the entire 2- to 17-year-old population of Seville or Spain.

The findings reported here, such as the high prevalence of caries and gingivitis, reflect the specific oral health conditions and access barriers encountered by minors residing in the studied areas of high social vulnerability. Consequently, these results should be interpreted as an indicator of the severe disease burden within these groups, rather than as a general population estimate.

Nonetheless, this study establishes a strong foundation for future research. Subsequent efforts should ideally utilize a population-based epidemiological design and a broader probabilistic sampling strategy to address these gaps and obtain representative provincial-level estimates. Validating these findings through more extensive studies will allow for more precise public health policy planning and the equitable allocation of resources in the region.

The study population was selected through the programs of the Luis Séiquer Social Dentistry Foundation [53,54], which is key to understanding the focus on the most vulnerable groups. Furthermore, its cross-sectional design prevents the establishment of causal relationships. The inclusion of country of origin as a categorical variable in our statistical analysis led to small sample sizes in some groups, which may have reduced the statistical robustness of those comparisons. Future research should consider grouping non-Spanish origins into broader categories and using longitudinal designs to better elucidate causal pathways.

The inclusion of country of origin as a categorical variable led SPSS to create separate categories for each non-Spanish country, some with very small sample sizes. This may have reduced the statistical robustness of these comparisons. In future research, grouping non-Spanish origins into broader categories would be advisable to improve interpretability and stability of the models.

However, certain limitations should be acknowledged. Additionally, the lack of systematic radiographic examination could have led to an underestimation of proximal caries. Moreover, as the study was based on a convenience sample of children from social programs, the findings may not be fully generalizable to the broader pediatric population. Its cross-sectional design prevents the establishment of causal relationships, and the fact that the research was conducted in a single geographical area may limit the generalizability of the findings. Future longitudinal studies are warranted to better elucidate causal pathways and to evaluate the effectiveness of school-based preventive interventions aimed at reducing oral health disparities.

### 4.7. Public Health Implications

Our findings underscore the urgent need for comprehensive public health policies aimed at reducing oral health disparities among vulnerable populations. This is particularly critical in the post-pandemic context, as factors such as limited access to care and changes in socio-behavioral habits may have exacerbated the oral health burden in disadvantaged groups [55]. Effective strategies should prioritize the implementation of early preventive programs targeting preschool-aged children, the development of culturally adapted oral health education tailored to migrant families, and the expansion of access to dental services through community-based initiatives. Furthermore, the integration of oral health surveillance into primary healthcare systems is essential for monitoring population needs and guiding interventions. Addressing these structural determinants is critical to achieving equitable oral health outcomes and reducing the long-term burden of untreated dental disease.

## 5. Conclusions

This study reveals a high prevalence of dental caries and substantial unmet treatment needs among children and adolescents from socially vulnerable populations in Seville, particularly in primary dentition. Poor oral health outcomes were strongly associated with sociodemographic and behavioral factors, including migrant background, low parental education, infrequent toothbrushing, and high sugar consumption, while fluoridated toothpaste use acted as a protective factor. These findings underscore the urgent need for early preventive strategies, culturally tailored oral health education, and equitable access to dental care to reduce disparities and improve oral health outcomes in vulnerable pediatric populations.:

## Figures and Tables

**Table 1:** Sociodemographic characteristics of the study population (*n* = 250).

Variable	Category	GA (2–6 Years, *n* = 37)	GB (7–11 Years, *n* = 107)	GC (12–17 Years, *n* = 106)	Total (*n* = 250)	Overall Mean ± SD
Age		5.08 ± 1.06	8.98 ± 1.43	14.98 ± 1.67	250	10.95 ± 3.99
Sex	Female	18 (48.6%)	49 (45.8%)	56 (52.8%)	123 (49.2%)	
	Male	19 (51.4%)	58 (54.2%)	50 (47.2%)	127 (50.8%)	
Country of Origin	Spanish	10 (27.0%)	49 (45.8%)	56 (52.8%)	115 (46.0%)	
	Latin American	14 (37.8%)	36 (33.6%)	31 (29.2%)	81 (32.4%)	
	African	8 (21.6%)	19 (17.8%)	17 (16.0%)	44 (17.6%)	
	Other	6 (16.2%)	3 (2.8%)	2 (1.9%)	11 (4.4%)	
School Grade	Kindergarten	19 (51.4%)	2 (1.9%)	0 (0.0%)	21 (8.4%)	
	Primary School	18 (48.6%)	105 (98.1%)	2 (1.9%)	125 (50.0%)	
	Secondary School	0 (0.0%)	0 (0.0%)	53 (50.0%)	53 (21.2%)	
	Upper Secondary	0 (0.0%)	0 (0.0%)	51 (48.1%)	51 (20.4%)	

**Table 2:** Pulp Therapy Needs by Age Group.

Group	*n*	Mean	SD
GA (2–6 years)	37	2.22	2.31
GB (7–11 years)	107	0.86	1.47
GC (12–17 years)	106	0.31	0.80

**Table 3:** Multivariate regression analysis of predictors of dental caries experience in the study population (*n* = 250).

Predictor (Reference)	B (Unstd.)	β (Std.)	95% CI (Lower–Upper)	*p*-Value
Decayed primary teeth (yes)	−0.754	−0.606	−0.892 to −0.616	<0.001
Missing primary teeth (yes)	−0.410	−0.157	−0.622 to −0.198	<0.001
Filled primary teeth (yes)	−0.876	−0.170	−1.288 to −0.464	<0.001
Gambia (yes)	−7.765	−0.123	−15.498 to −0.033	0.049
Palestine (yes)	−8.765	−0.139	−16.498 to −1.033	0.026
Ukraine (yes)	−4.265	−0.189	−7.080 to −1.450	0.003
Educational level: Kindergarten (yes)	−4.108	−0.286	−4.769 to −3.447	<0.001
Brushing frequency: once per day (yes)	−1.584	−0.193	−2.662 to −0.507	0.004
Frequent sugar consumption (yes)	−2.945	−0.237	−4.456 to −1.434	<0.001
Need for pulp therapy: 1 tooth	−1.822	−0.146	−3.299 to −0.345	0.016
Need for pulp therapy: 3 teeth	−4.391	−0.216	−6.780 to −2.002	<0.001
Need for pulp therapy: 4 teeth	−4.891	−0.241	−7.280 to −2.502	<0.001
Need for pulp therapy: 6 teeth	−5.091	−0.179	−8.421 to −1.761	0.003
Need for pulp therapy: 8 teeth	−8.391	−0.188	−13.610 to −3.172	0.002
DMFT Index	0.203	0.186	0.089 to 0.316	0.001
Educational level: Middle school (yes)	5.047	0.518	4.588 to 5.507	<0.001
Educational level: High school (yes)	7.799	0.789	7.334 to 8.265	<0.001
Flossing (yes)	1.860	0.223	0.841 to 2.880	<0.001

Model statistics: R = 0.814; adjusted R^2^ = 0.637; *p* < 0.001; 95% CI for the model = 11.343–13.807.

## Data Availability

The raw data supporting the conclusions of this article will be made available by the authors on request.
